# Supplementary material for: Investigating the Vascular Toxicity Outcomes of the Irreversible Proteasome Inhibitor Carfilzomib
Source: Int J Mol Sci. 2020 Jul 22;21(15):5185. doi: 10.3390/ijms21155185 (PMC7432349; doi:10.3390/ijms21155185)
Supplement: Supplementary file 1 [file ijms-21-05185-s001.pdf]

Investigating the Vascular Toxicity Outcomes of the Irreversible Proteasome Inhibitor Carfilzomib

Panagiotis Efentakis [1,2], Hendrik Doerschmann [2], Claudius Witzler [2], Svenja Siemer [3], Panagiota-Efstathia Nikolaou [1], Efstathios Kastritis [4], Roland Stauber [3], Meletios Athanasios Dimopoulos [4], Philip Wenzel [2,5,6,†], Ioanna Andreadou [1,*,†] and Evangelos Terpos [4,†]

[1] Laboratory of Pharmacology, Faculty of Pharmacy, National and Kapodistrian University of Athens, 15771 Athens, Greece; pefentakis@pharm.uoa.gr (P.E.); nayanik@pharm.uoa.gr (P.-E.N.)

[2] Cardiology I Department, University Medical Center of the Johannes Gutenberg-University Mainz, 55131 Mainz, Germany; h.doerschmann@uni-mainz.de (H.D.); c.witzler@uni-mainz.de (C.W.); wenzelp@uni-mainz.de (P.W.)

[3] Molecular and Cellular Oncology/ENT, University Medical Center of the Johannes Gutenberg-University Mainz, Langenbeckstr. 1, 55101 Mainz, Germany; svenja.siemer@uni-mainz.de (S.S.); rstauber@uni-mainz.de (R.S.)

[4] Department of Clinical Therapeutics, School of Medicine, National and Kapodistrian University of Athens, 11528 Athens, Greece; ekastritis@gmail.com (E.K.); mdimop@med.uoa.gr (M.A.D.); eterpos@med.uoa.gr (E.T.)

[5] Center for Cardiology—Cardiology I, University Medical Center Mainz, Langenbeckstraße 1, 55101 Mainz, Germany

[6] German Center for Cardiovascular Research (DZHK), Partner Site Rhine-Main, Germany

† These authors contributed equally to this work.

* Correspondence: jandread@pharm.uoa.gr

Supplemental Figures

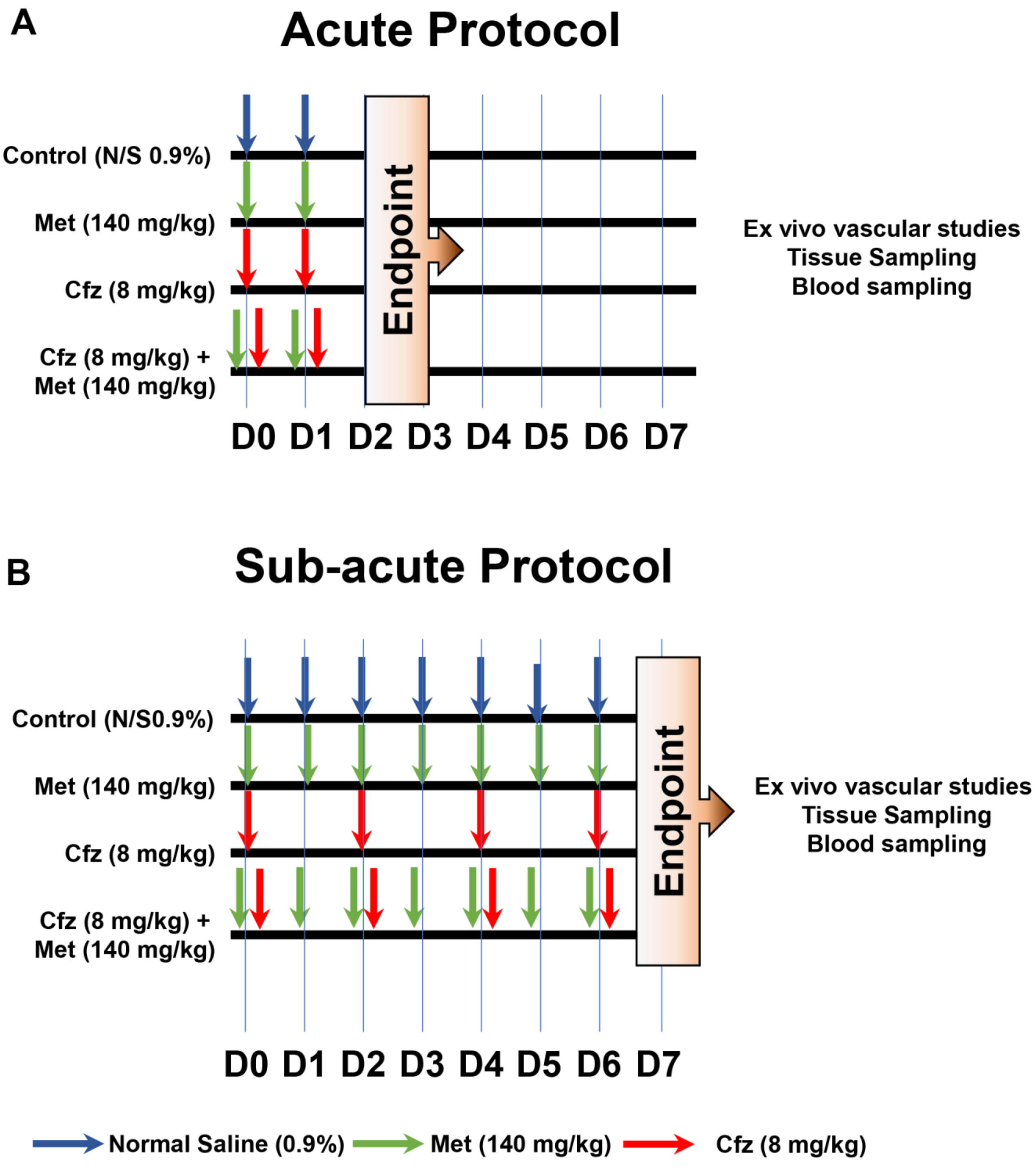

Figure S1. Representative workflows of in vivo experiments. Timepoints and endpoints of the **A.** acute and **B.** sub-acute in vivo protocols. Blue lines represent normal saline (N/S 0.9%) used as vehicle in the experiments; green lines represent metformin per os administration (Met, 140 mg/kg); red lines represent carfilzomib intraperitoneal administration (Cfz, 8mg/kg).

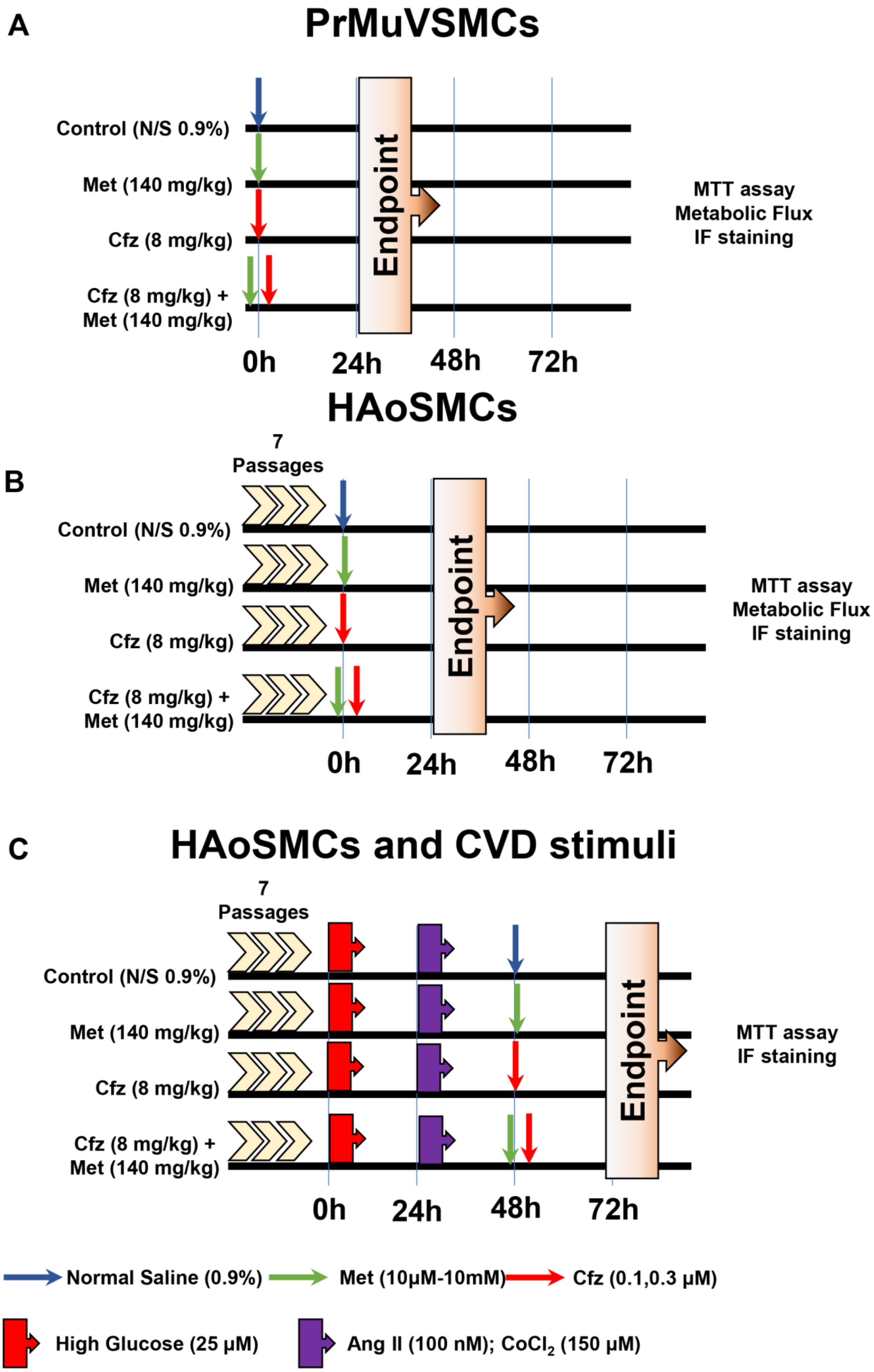
A
PrMuVSMCs
Control (N/S 0.9%)
Met (140 mg/kg)
Cfz (8 mg/kg)
Cfz (8 mg/kg) +
Met (140 mg/kg)
Endpoint
MTT assay
Metabolic Flux
IF staining
0h
24h
48h
72h
HAoSMCs
B
7
Passages
Control (N/S 0.9%)
Met (140 mg/kg)
Cfz (8 mg/kg)
Cfz (8 mg/kg) +
Met (140 mg/kg)
Endpoint
MTT assay
Metabolic Flux
IF staining
0h
24h
48h
72h
C
HAoSMCs and CVD stimuli
7
Passages
Control (N/S 0.9%)
Met (140 mg/kg)
Cfz (8 mg/kg)
Cfz (8 mg/kg) +
Met (140 mg/kg)
Endpoint
MTT assay
IF staining
0h
24h
48h
72h
Normal Saline (0.9%)
Met (10μM-10mM)
Cfz (0.1,0.3 μM)
High Glucose (25 μM)
Ang II (100 nM); CoCl2 (150 μM)

Figure S2. Representative workflows of the in vitro experiments. Timepoints and endpoints of the **A.** Primary murine vascular smooth muscles (PrmVSMCs), **B.** Human aortic smooth muscle cells (HAoSMCs) and **C.** Human aortic smooth muscle cells (HAoSMCs) treated with cardiovascular disease (CVD) stimuli. Blue lines represent normal saline (N/S 0.9%) used as vehicle in the experiments; green lines represent metformin per os administration (Met, 140 mg/kg); red lines represent carfilzomib intraperitoneal administration (Cfz, 8mg/kg). Red arrowed boxes represent high glucose administration (25 μM), while purple arrowed boxes represent angiotensin II (Ang II, 100 nM) or cobalt chloride ($CoCl_2$, 150 μM) administration.

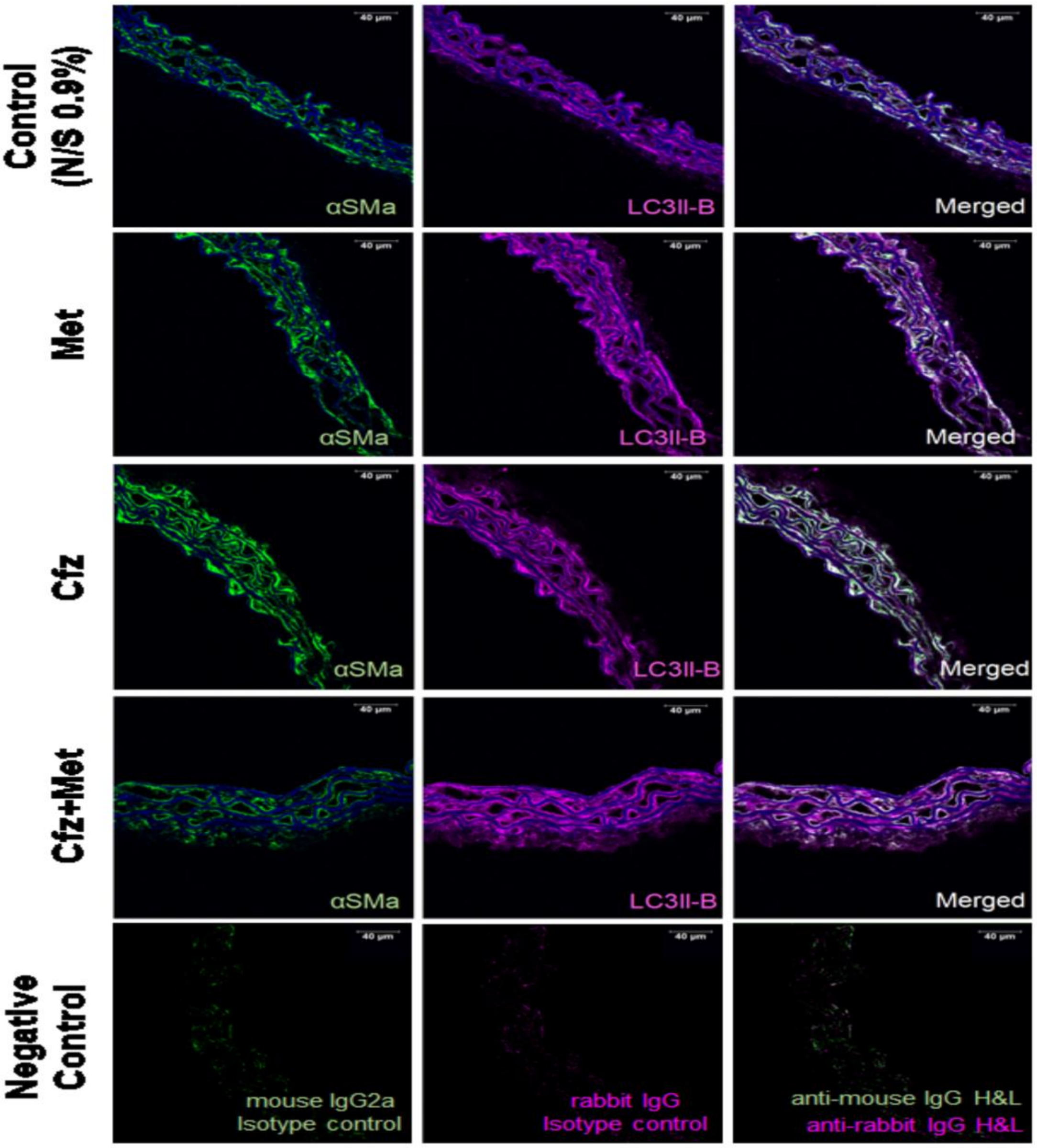

Figure S3. Carfilzomib and metformin synergistically increase LC3-B expression and downregulate αSMA in murine aortas. Representative immunofluorescent images originating from the confocal microscopy of murine aortas stained against α-smooth muscle actin (αSMA; green) and LC3II-B (magenta), as well as the merged fluorescent images of the green and magenta channel (white). Negative controls of the staining using mouse and rabbit isotype controls and anti-mouse and anti-rabbit IgG H&L secondary antibodies.

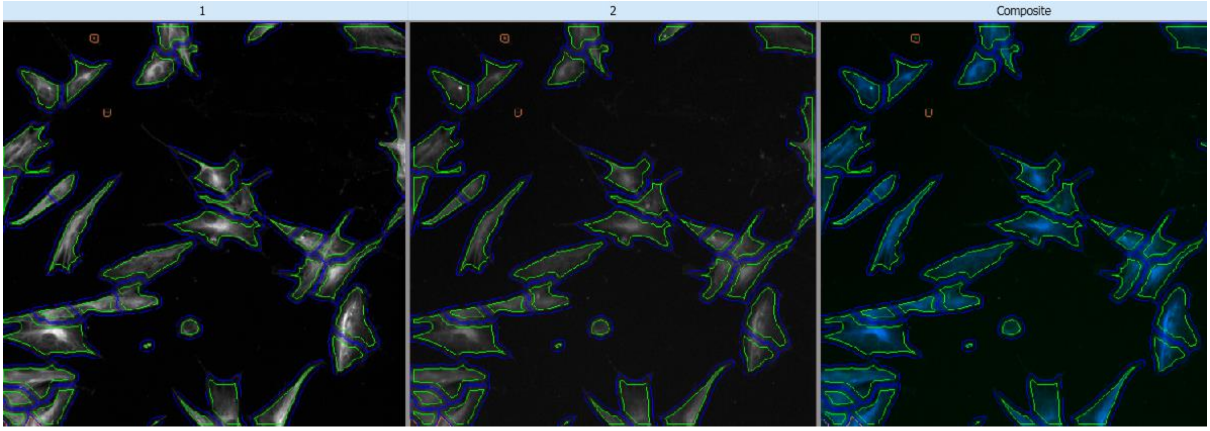

Figure S4. Vascular smooth muscle cell masks selected for automated microscopy. Representative images of the masks selected for automated microscopy. Channel 1 represents DAPI signal and Channel 2 represents LC3-B signal, while channel 3 represents the merged (composite) image. .
